# Supplementary material for: Conserved interactions required for inhibition of the main protease of severe acute respiratory syndrome coronavirus 2 (SARS-CoV-2)
Source: Sci Rep. 2020 Nov 30;10:20808. doi: 10.1038/s41598-020-77794-5 (PMC7704658; doi:10.1038/s41598-020-77794-5)
Supplement: Supplementary file 1 — Supplementary Information. [file 41598_2020_77794_MOESM1_ESM.pdf]

Supplementary file:

Conserved interactions required for inhibition of the main protease of severe acute respiratory syndrome coronavirus 2 (SARS-CoV-2)

Alina Shitrit, Daniel Zaidman, Ori Kalid, Itai Bloch, Dvir Doron, Tali Yarnizky, Idit Buch, Idan Segev, Efrat Ben-Zeev, Elad Segev and Oren Kobiler

Supplementary Table 1

Drug name	Structure	Smiles	Glide/ GOLD	Avg Inh. (%)
**GSK-256066**	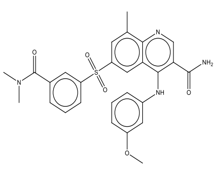	CN(C)C(=O)c1cc(ccc1)S(=O)(=O)c(c2)cc(C)c(c23)ncc(C(=O)N)c3Nc4cc(OC)ccc4	GOLD	36.96
**AVANAFIL**	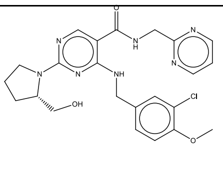	n1cccnc1CNC(=O)c2c(NCc3cc(Cl)c(cc3)OC)nc(nc2)N4CCC[C@H]4CO	GOLD	12.24
**LAMIVUDINE**	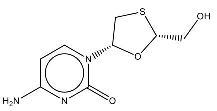	OC[C@@H]1O[C@@H](CS1)n(c(n2)=O)ccc2N	Glide	9.29
**BIMIRALISIB**	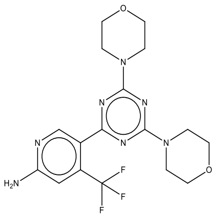	c1c(N)ncc(c1C(F)(F)F)-c2nc(N3CCOCC3)nc(n2)N4CCOCC4	GOLD	5.97
**MELOXICAM**	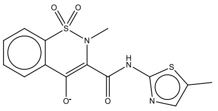	Cc1cnc(s1)NC(=O)C(=C2[O-])N(C)S(=O)(=O)c(c23)cccc3	GOLD	4.82
**VOXTALISIB**	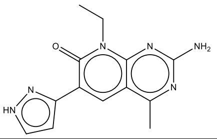	Cc1nc(N)nc(c12)n(CC)c(=O)c(c2)-c3cc[nH]n3	GOLD	4.38
**BENZNIDAZOLE**	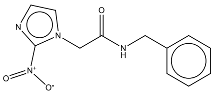	[O-][N+](=O)c1nccn1CC(=O)NCc2ccccc2	GOLD	4.09
**TAK-715**	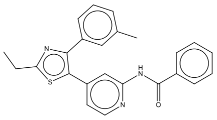	c1ccccc1C(=O)Nc(ncc2)cc2-c(sc(n3)CC)c3-c(cc4C)ccc4	Both	2.47
**DARUSENTAN**	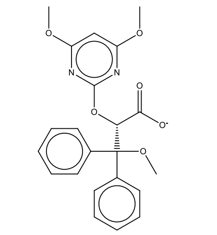	c1ccccc1C(OC)(c2ccccc2)[C@@H](C([O-])=O)Oc(n3)nc(OC)cc3OC	Both	2.42
**ERLOTINIB**	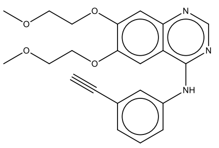	C#Cc1cc(ccc1)Nc2ncnc(c23)cc(OCCOC)c(c3)OCCOC	Glide	2.10

**TAMOXIFEN CITRATE**	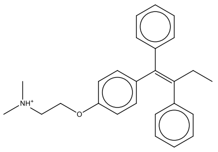	c1ccccc1C(\CC)=C(c2ccccc2)/c3ccc(cc3) OCC[NH+](C)C	GOLD	1.61
**SOLCITINIB**	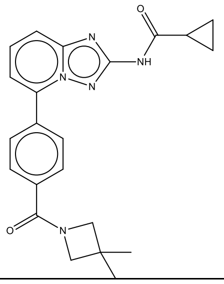	C1CC1C(=O)Nc(n2)nn(c23)c(ccc3)-c(cc4)ccc4C(=O) N(C5)CC5(C)C	GOLD	0.84
**PAGOCLONE**	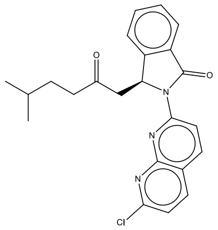	CC(C)CCC(=O)C[C@@H](c(c12)cccc2)N(C1=O)c(cc3)nc(c34)nc(Cl)cc4	GOLD	<0
**VIPADENANT**	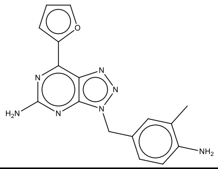	c1cc(N)c(C)cc1Cn(nn2)c(c23)nc(N)nc3-c4ccco4	GOLD	<0
**VADADUSTAT**	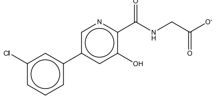	[O-]C(=O)CNC(=O)c1c(O)cc(cn1)-c2cc(Cl)ccc2	GOLD	<0
**CEPHALEXIN**	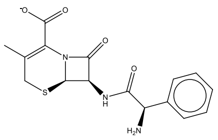	c1ccccc1[C@@H](N)C(=O)N[C@@H]2C(=O)N([C@@H]23)C(C([O-])=O)=C(C)CS3	Glide	<0
**ARMODAFINIL (MBX-102)**	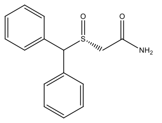	C1=CC=C(C=C1)C(C2=CC=CC=C2)[S@](=O)CC(=O)N	Glide	<0
**TERIFLUNOMIDE**	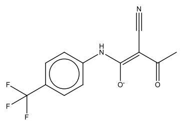	CC(=O)C(\C#N)=C([O-])\Nc(cc1)ccc1C(F)(F)F	Glide	<0
**BETRIXABAN**	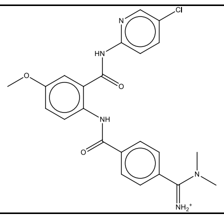	CN(C)C(=[NH2+])c1ccc(cc1)C(=O)Nc(ccc(c2)OC)c2C(=O)Nc(nc3)ccc3Cl	GOLD	<0
**RIBOCICLIB**	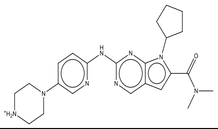	C1CCCC1n(c(c2)C(=O)N(C)C)c(c23)nc(nc3)Nc(nc4)ccc4N5CC[NH2+]CC5	GOLD	<0

**SELICICLIB**	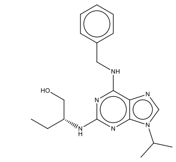	CC(C)n(cn1)c(c12)nc(N[C@H](CC)CO)nc2NCc3ccccc3	Both	<0
**LAROTRECTINIB**	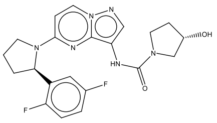	O[C@H]1CCN(C1)C(=O)Nc2cnn(c23)ccc(n3)N4CCC[C@@H]4c5c(F)ccc(F)c5	Glide	<0
**GLAFENINE**	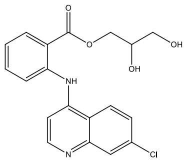	C1=CC=C(C(=C1)C(=O)OCC(CO)O)NC2=C3C=CC(=CC3=NC=C2)Cl	Both	<0
**TG100-115**	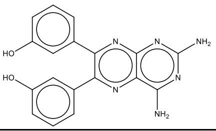	Nc1nc(N)nc(c12)nc(-c3cc(O)ccc3)c(n2)-c4cc(O)ccc4	GOLD	<0
**DAROLUTAMIDE**	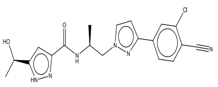	N#Cc1c(Cl)cc(cc1)-c2ccn(n2)C[C@H](C)NC(=O)c3cc([nH]n3)[C@@H](C)O	Glide	<0
**REBAMIPIDE**	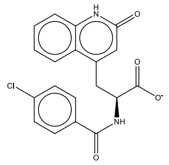	c1cc(Cl)ccc1C(=O)N[C@H](C([O-])=O)Cc2cc(=O)[nH]c(c23)cccc3	GOLD	<0
**LONIDAMINE**	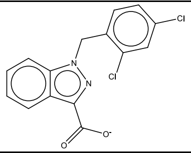	c1cccc(c12)n(nc2C([O-])=O)Cc3c(Cl)cc(Cl)cc3	Glide	<0
**GANDOTINIB**	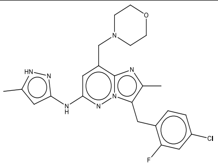	c1cc(Cl)cc(F)c1Cc(c(n2)C)n(c23)nc(Nc(n[nH]4)cc4C)cc3CN5CCOCC5	GOLD	<0
**RIBOFLAVIN**	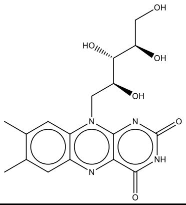	O=c1[nH]c(=O)nc(c12)n(C[C@H](O)[C@H](O)[C@H](O)CO)c3c(n2)cc(C)c(C)c3	Glide	<0

Supplementary Table 2

Drug name	Structure	Smiles	Covalent moiety	Avg Inh.
**Bicalutamide**	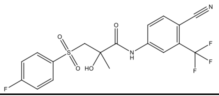	CC(O)(CS(=O)(=O)C1=CC=C(F)C=C1)C(=O)NC1=CC(=C(C=C1)C#N)C(F)(F)F	Nitril	35.01
**Ruxolitinib**	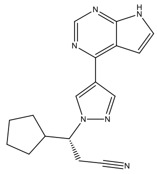	N#CC[C@H](C1CCCC1)N1C=C(C=N1)C1=C2C=CNC2=NC=N1	Nitril	19.23
**Trilostane**	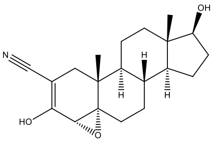	[H][C@@]12CC[C@H](O)[C@@]1(C)CC[C@@]1([H])[C@@]2([H])CC[C@@]23O[C@@H]2C(O)=C(C[C@]13C)C#N	Nitril	0.37
**Anastrozole**	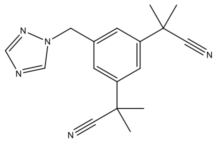	CC(C)(C#N)C1=CC(=CC(CN2C=NC=N2)=C1)C(C)(C)C#N	Nitril	4.07
**WP1066**	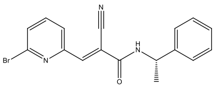	C[C@H](NC(=O)C(=C\C1=CC=CC(Br)=N1)\C#N)C1=CC=CC=C1	Michael Acceptors	<0
**Tofacitinib**	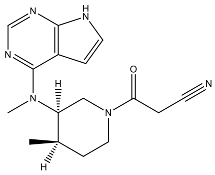	[H][C@@]1(C)CCN(C[C@]1([H])N(C)C1=NC=NC2=C1C=CN2)C(=O)CC#N	Nitril	<0

Supplementary Table 3

Structure	Smiles	Name as in reference	IC50 (μM) as in reference	Avg Inh. at 20μM	Avg Inh. at 50μM	IC50 (μM)	Relative Solubility at 20mM
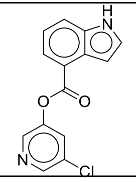	O=C(c(ccc1)c2c1[nH]cc2)Oc3cc(Cl)cnc3	10_GRL-0496[1]	0.03	99.84	100.01	0.05	0.99
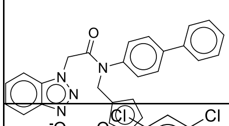	O=C(N(c1ccc(c2ccccc2)cc1)Cc3cscc3)Cn(nn4)c5c4cccc5	17a[1]	0.051	30.41	39.14	5.32	0.64
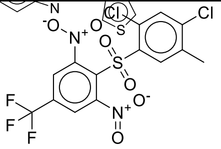	Cc1c(Cl)cc(Cl)c(S(=O)(c2c([N+]([O-])=O)cc(C(F)(F)F)cc2[N+]([O-])=O)=O)c1	3[2]	0.3		9.61		
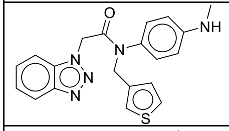	CNc1ccc(N(C(Cn(nn2)c3c2cccc3)=O)Cc4cscc4)cc1	16j[3]	2.1	57.12	82.62	4.33	0.99
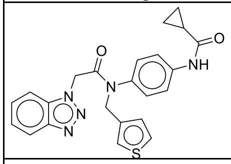	O=C(C1CC1)Nc2ccc(N(C(Cn(nn3)c4c3cccc4)=O)Cc5cscc5)cc2	16e (ML300)[3]	4.11	68.97	70.20	3.99	0.90
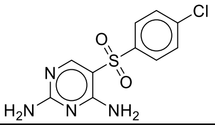	Nc1nc(N)c(S(=O)(c2ccc(Cl)cc2)=O)cn1	5[2]	6		-22.07		
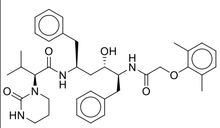	Cc1c(OCC(N[C@H]([C@H](C[C@@H](NC([C@@H](N2CCCNC2=O)C(C)C)=O)Cc3ccccc3)O)Cc4ccccc4)=O)c(C)ccc1	Lopinavir[4]	>32	7.22	12.97		0.83

1 Ghosh, A. K. *et al.* Design, synthesis and antiviral efficacy of a series of potent chloropyridyl ester-derived SARS-CoV 3CLpro inhibitors. *Bioorg Med Chem Lett* **18**, 5684-5688, doi:10.1016/j.bmcl.2008.08.082 (2008).

2 Lu, I. L. *et al.* Structure-based drug design and structural biology study of novel nonpeptide inhibitors of severe acute respiratory syndrome coronavirus main protease. *J Med Chem* **49**, 5154-5161, doi:10.1021/jm060207o (2006).

3 Turlington, M. *et al.* Discovery of N-(benzo[1,2,3]triazol-1-yl)-N-(benzyl)acetamido)phenyl) carboxamides as severe acute respiratory syndrome coronavirus (SARS-CoV) 3CLpro inhibitors: identification of ML300 and noncovalent nanomolar inhibitors with an induced-fit binding. *Bioorg Med Chem Lett* **23**, 6172-6177, doi:10.1016/j.bmcl.2013.08.112 (2013).

4 de Wilde, A. H. *et al.* Screening of an FDA-approved compound library identifies four small-molecule inhibitors of Middle East respiratory syndrome coronavirus replication in cell culture. *Antimicrob Agents Chemother* **58**, 4875-4884, doi:10.1128/AAC.03011-14 (2014).
