# Supplementary material for: Conserved interactions required for inhibition of the main protease of severe acute respiratory syndrome coronavirus 2 (SARS-CoV-2)
Source: Sci Rep. 2020 Nov 30;10:20808. doi: 10.1038/s41598-020-77794-5 (PMC7704658; doi:10.1038/s41598-020-77794-5)
Supplement: Supplementary file 2 — Supplementary Legends. [file 41598_2020_77794_MOESM2_ESM.docx]

**Supplementary Table Legends**

**Supplementary Table 1: Non-covalent compounds that were selected for testing and their % of inhibition at 50μM concentration.** A list of all non-covalent compounds tested in the protease inhibition assay after selection either by the GOLD, Glide or both docking tools. Percent average inhibition at 50μM is presented (Avg. Inh).

**Supplementary Table 2: Covalent compounds that were selected for testing and their % of inhibition at 50μM concentration.** A list of all covalent compounds tested in the protease inhibition assay after selection. Percent average inhibition at 50μM is presented (Avg. Inh).

**Supplementary Table 3: Several controls of known SARS-CoV-1 Mpro inhibitors and their results in the protease inhibition assay.** The structures (column A) and activity Column C) of several SARS-CoV-1 inhibitors are known from the publications (column D) along with the results obtained in our protease inhibition assay (columns E-H).
